# Determination of the effect of alkaline chemical modification using sodium hydroxide on the acoustic and thermal properties of bagasse fibers

**DOI:** 10.1038/s41598-025-02385-1

**Published:** 2025-05-20

**Authors:** Roohalah Hajizadeh, Amirhossein Montazeri, Mehrana Esnaasharieh, Mohammad Hossein Beheshti, Zeinab Garavand

**Affiliations:** 1 Department of Occupational Health and Safety, School of Public Health and Safety, Shahid Beheshti University of Medical Sciences, Tehran, Iran; 2 School of Health, Social Determinants of Health Research Center, Gonabad University of Medical Sciences, Khorasan Razavi, Iran; 3 School of Public Health, Zanjan University of Medical Sciences, Zanjan, Iran

**Keywords:** Bagasse fibers, Chemical modification, Sodium hydroxide, Sound absorption coefficient, Thermal insulation, Environmental sciences, Planetary science, Health occupations, Engineering, Materials science

## Abstract

Natural fibers have gained attention as sustainable alternatives due to their environmental advantages, sound absorption capability and thermal insulation properties. This study investigates alkaline treatment to improve the acoustic and thermal behavior of bagasse fibers for sustainable insulation applications. Chemical modification through alkaline treatment was performed using sodium hydroxide at concentrations of 1–4% and immersion times ranging from 2 to 24 h. Experimental designs were based on a factorial approach, and the data were analyzed using Design Expert software (version 11). Additionally, after conducting the experiments, analysis of variance (ANOVA) was used to develop models describing the relationships between the investigated parameters and response variables to predict optimal conditions. The results indicated that the concentration of sodium hydroxide and immersion time significantly influenced the fiber structure and its sound absorption coefficient. Specifically, a 3% concentration and 8-hour immersion time improved the sound absorption coefficient, whereas concentrations above 4% caused fiber deformation due to excessive reaction. Additionally, the peak sound absorption was observed in the frequency range of 1000 to 2000 Hz. The impact of alkaline treatment on thermal conductivity was minimal, with values ranging from 0.091 to 0.127 W/(m^2^ K). Although the reference value for effective thermal insulators is typically considered to be 0.065 W/(m^2^ K), and the measured values for treated bagasse were higher than this threshold, they remain too small to qualify bagasse as a strong thermal insulator. However, alkaline treatment increased the Sound Absorption Average (SAA) by 94.11% at low frequencies, 18.44% at mid frequencies, and 35.3% at high frequencies. Considering the economic and environmental advantages of these fibers and their growing application in polymer composites, this optimized process serves as an efficient method to enhance the compatibility of natural fibers with polymer matrices and improve the acoustic and mechanical properties of building composites.

## Introduction

In recent decades, the increase in awareness about sustainable development and environmental protection has brought attention to the use of renewable resources and agricultural waste as sustainable alternatives to synthetic materials^1,2^. Natural fibers, due to their unique properties and easy accessibility, have gained special attention in various industries, including the construction industry^3^. Incorporating plantain peel and ramie-luffa fibers into bio-based composites has shown notable improvements in tensile strength, water resistance, and biodegradability during storage. These findings support the importance of fiber selection and treatment, which this study extends by optimizing the acoustic and thermal performance of alkali-treated bagasse fibers^4,5^. Chemical surface treatments have been shown to significantly improve the performance of natural fibers. Alkaline–silane modification has enhanced the thermal stability of Hibiscus fibers, while liquid smoke treatment improved the tensile strength and crystallinity of Sansevieria fibers. These support the potential of using NaOH-treated bagasse, as investigated in this study, for thermal and acoustic applications^6,7^. One of the agricultural wastes with high potential is bagasse, a byproduct of sugarcane processing. This material, rich in cellulose and lignin, is considered an ideal option for producing sustainable sound and thermal insulators^8^. Recent studies demonstrate that natural fibers can be effectively transformed into eco-friendly acoustic and thermal insulation materials. These materials exhibit excellent sound absorption and insulation properties, with optimized density and thickness enhancing their performance as validated by advanced predictive models. Such advancements not only mitigate noise pollution and improve energy efficiency but also promote the sustainable use of renewable resources in modern construction^9–12^. The chemical processing and modification of bagasse, especially through alkaline methods, can enhance its properties and enable its use in diverse applications in the construction industry. Alkali–silane treatment of waru bark fibers has shown significant improvements in crystallinity (63.02%) and tensile strength (243.94 MPa), reinforcing their potential in polymer composites—an approach that aligns with the NaOH-based treatment explored in this study^13^. Nowadays, the industrialization of societies has caused the workforce to encounter equipment that can pose various health threats. One of these threats is noise pollution^11^ caused by the operation of machinery and equipment, which has become a problem in various spaces, particularly in-built environments such as buildings and industrial facilities. Sound, as a harmful physical factor, can have many negative impacts on individuals^14^. These impacts include hearing impairment, reduced concentration, increased stress, cardiovascular problems, and sleep disorders^15–17^. In industrial environments and residential and commercial buildings, in addition to existing sound sources, the reflection of sound from various surfaces can also increase the intensity of noise pollution^18,19^.

Prolonged exposure to noise, especially in noisy industrial environments, creates numerous problems that not only lead to hearing loss but can also increase accidents, absenteeism, and reduce the productivity of exposed workers^20^. Research has shown that exposure to noise, particularly at specific frequencies, can have severe negative effects on the health and well-being of individuals^21^. These effects may include mental and cognitive disorders, reduced workplace productivity, and an increase in industrial accidents^22^.

To mitigate these negative impacts, the use of various sound control materials and techniques, including sound absorbers, has gained significant importance^23,24^. To reduce noise pollution and manage workplace noise, various methods are available, including technical and engineering controls, administrative controls (such as exposure time management), training, and awareness-raising^25^. One effective method for noise pollution control involves the use of porous materials and resonant-based sound absorbers^26,27^. Porous materials are particularly effective at absorbing sound at mid-to-high frequencies. Due to their internal structure, which contains tiny pores, these materials can absorb acoustic energy through thermal and viscous losses^28^. The fundamental principle of these absorbers is internal resonance effects^29^ which enable them to achieve good sound absorption coefficients at low frequencies, although their sound absorption frequency range is typically limited^30,31^. Porous materials, containing numerous pores, dissipate the energy of sound waves entering them through internal friction via thermal and viscous losses. Due to this principle, porous materials generally exhibit sound absorption across a broader frequency range^32,33^.

Porous materials are classified into three categories based on their microscopic structure: cellular, fibrous, and granular^34^. Currently, natural materials such as plant fibers, wood, jute, and hemp have emerged as suitable options for sound absorbers. Due to characteristics like low density, suitable mechanical properties, easy accessibility, and cost-effectiveness, these materials are gradually replacing synthetic materials such as fiberglass and polyester. Moreover, the use of these materials in buildings can help reduce environmental impacts, enhance the quality of living and working environments, and contribute to sustainability^35^ .

The use of natural and recycled materials is not only economically viable but also has significantly lower environmental impacts. Natural materials regulate indoor air humidity, and their distinctive aroma has a positive psychological effect on humans. An experiment conducted in a straw house in Germany, where several important features were systematically measured, demonstrated promising results for healthy living conditions^36^. Many of these materials are readily available as agricultural or industrial waste and can be easily utilized in the production of sound absorbers. In this regard, various studies have been conducted on the application of natural fibers such as straw, reeds, hemp, and even industrial waste like tea leaves and sugarcane bagasse.

In recent years, natural fibers have been specifically considered as reliable materials for producing sound-absorbing panels at lower costs^37^ These fibers often possess excellent sound and thermal insulation properties^38^, have no harmful effects on health, are widely available, and are frequently produced as waste from other manufacturing processes. Several studies have investigated the role of plants and leaves as sound absorbers^39,40^. With the growing interest in using natural and recycled materials, as well as by-products from other production processes, the use of green and sustainable products with low environmental impact in the construction industry is increasing.

Given the wide variety of these materials across different countries, further research is needed to thoroughly examine their acoustic and thermal behavior. In Iran, due to the abundance of agricultural waste such as wool and sugarcane bagasse, these materials can serve as valuable resources for producing sound absorbers and thermal insulators. Utilizing these resources can not only mitigate environmental issues but also significantly reduce production costs.

Natural fibers, due to their unique properties, easy accessibility, and environmental compatibility, have gained attention as sustainable alternatives to synthetic materials in controlling noise pollution. One such material is bagasse, a byproduct of sugarcane processing, which, with its rich cellulose and lignin content, demonstrates high potential for producing efficient sound absorbers. Utilizing agricultural waste like bagasse not only reduces agricultural waste but also leads to the development of innovative materials for managing noise and thermal pollution.

Natural sound absorbers, leveraging the physical and chemical properties of agricultural fibers, can help reduce sound reflections and improve acoustic quality in industrial and building environments. The chemical and mechanical modification of these materials, including through alkaline treatment methods, can enhance their sound absorption and thermal insulation properties. These methods not only improve material efficiency but also reduce dependency on non-renewable resources.

The present study aims to evaluate and enhance the performance of modified bagasse fibers in producing sound absorbers and thermal insulators for building environments. This approach represents an effective step toward reducing environmental impacts, improving sustainability in the construction industry, and increasing the productivity of agricultural materials. By focusing on the potential of natural fibers, this research seeks to offer optimized solutions for utilizing renewable resources in managing noise and thermal pollution. Unlike previous studies that have typically addressed either the acoustic or thermal characteristics of natural fibers separately, this study presents a combined experimental and statistical approach to evaluate both properties simultaneously in sodium hydroxide-treated bagasse fibers.

## Methodology

### Sample preparation

Bagasse, a dry fibrous residue obtained from the sugarcane refining process, is extracted in the form of small, compressed chips. In this study, the bagasse used was sourced from the Sugarcane Cultivation and Development Research Center in Khuzestan Province, Iran, and transferred to the laboratory approximately three months after harvest, following an initial natural drying period. Bagasse was first converted into fine particles in two stages using a shredder and then dried at ambient temperature for 15 days. Afterward, the shredded bagasse was sieved to separate wood fragments of suitable dimensions, which were stored in plastic bags to prevent moisture absorption.

### Chemical treatment procedure

In this study, an alkaline treatment method using sodium hydroxide (manufactured by Merck, Germany) was employed as one of the common chemical modification techniques for natural bagasse fibers. The experiments were designed following a factorial design approach based on two main parameters: sodium hydroxide concentration and immersion time. The experiments were conducted on 50gram bagasse samples with initial sodium hydroxide concentrations of 2, 4, 6, 8, and 10% and immersion times of 2, 8, 13, 18, and 24 h. Laboratory tests revealed that using a combination of sodium hydroxide and PVA adhesive (manufactured by Sigma company) at concentrations above 4% resulted in the destruction of the bagasse fiber structure, making it impossible to prepare suitable samples. To prepare sodium hydroxide solutions at concentrations of 1, 2, 3, and 4%, the precise amount of sodium hydroxide was weighed using a precise Prezisa scale and the SMA-FR 262 model from Switzerland, and then dissolved in 1 L of distilled water; to ensure complete dissolution, the solution was stirred with a magnetic stirrer at 80 °C for 3 h. Subsequently, the 50gram bagasse samples were immersed in the specified solutions for the designated times, and after the immersion period, the bagasse was filtered and dried at ambient temperature for 20 days. Finally, to produce samples suitable for acoustic and thermal testing, the treated bagasse was bonded with polyvinyl alcohol adhesive into molds of specified dimensions, and after 10 days of final drying at ambient temperature, the samples were sent to the acoustic and thermal laboratory.

### Composite fabrication

**Fig. 1:**
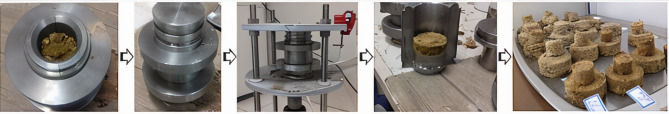
The process of preparing samples for acoustic testing.

The required amount of bagasse for each specimen was calculated based on the target density and thickness, and the fiber–binder mixture was thoroughly blended before molding. As illustrated in Fig. 1, to prepare samples for acoustic and thermal testing, the treated bagasse was molded into specimens with diameters of 3 cm and 6 cm—matching the impedance tube dimensions—and bonded with PVA adhesive under pressure for 4 h. The samples were then dried at room temperature (25–30 °C) and transferred to the laboratory for further testing of their acoustic and thermal properties. A 6 wt% solution of polyvinyl alcohol (PVA) was used as a binder relative to the dry mass of bagasse.

### Sound absorption measurement

To evaluate the acoustic behavior of natural bagasse fiber composites during alkaline treatment and compare them with raw fibers, the sound absorption coefficient was measured. The measurement was performed using a BSWA impedance tube device (model SW360) in accordance with ISO 10534-2 standards. This apparatus, featuring tubes with 3 cm and 6 cm diameters, enabled accurate measurement of sound absorption and transmission loss across various frequencies. The device operates by coupling a sound source to one end of the tube while the test sample is mounted at the other end, making it suitable for assessing both the absorption properties and surface acoustic impedance of porous materials. For low-frequency measurements (100–1600 Hz), a 6 cm diameter tube was utilized, whereas a 3 cm tube was used for high-frequency ranges (1600–6300 Hz).

### Thermal conductivity measurement

Thermal conductivity was measured using a Decagon KD2 Pro thermal analyzer at the Par Tavous Research Institute in Mashhad. This handheld device, equipped with KS-1 single-needle and dual-needle sensors, allowed for the determination of thermal conductivity, resistance, specific heat capacity, and diffusivity over a specified range. Samples were fabricated as cylinders with a diameter of 10 cm and a height of 7 cm, then dried under standard conditions until the weight difference between successive drying stages was less than 0.1 g, ensuring consistency in thermal measurements.

### Airflow resistance measurement

Airflow resistance, a key parameter influencing the sound absorption of porous materials, was measured according to the ASTM C522-3 standard. In this test, by varying the airflow speed between 5 and 50 mm/s, the pressure drop (∆p) across the samples was recorded using a differential manometer, and the airflow resistance was calculated using the Eq. (1).

1 $$\sigma =\frac{{\vartriangle \rho }}{{vt}}$$

### Surface density measurement

Surface density (mass per unit area) was determined according to ASTM D3776 using a digital balance (Shimadzu, Japan). Each sample was measured ten times to ensure accuracy.

### Surface porosity and pore size measurement

Surface porosity was calculated by analyzing SEM images with MATLAB (Version 7) using image processing algorithms. The SEM images were converted into binary images (black and white) through thresholding, with pixel values of 0 representing black and 255 representing white, from which the porosity percentage was determined. Pore size was measured using ImageJ software.

### Variables and experimental design

A full factorial design was used for optimization. The two independent variables were NaOH concentration (A) and immersion time (B). Dependent variables included SAC across frequency bands, thermal conductivity, porosity of fiber’s surface, and airflow resistance. Statistical analyses, including ANOVA and RSM modeling, were performed using Design Expert software (v11).

### Replication and randomization

All tests were performed in triplicate to ensure accuracy and reproducibility. Samples were randomly assigned to treatment conditions to minimize systematic errors.

## Results

### Bagasse composite acoustics: thickness and NaOH effects

The sound absorption coefficient of composites made from bagasse with a density of 150 kg/m^3^ and varying thicknesses (1 to 5 cm) was measured using the impedance tube device. Results indicate that as the thickness of the sound-absorbing material increases, the peak frequency shifts to lower frequencies. Therefore, increasing the thickness of the sound-absorbing material enhances its performance in lower frequencies. According to the findings, the maximum sound absorption coefficient of bagasse composites occurs in the frequency range of 1000 to 2000 Hz. The noise reduction coefficients (NRC) for bagasse composites with thicknesses of 1, 2, 3, 4, and 5 cm were 0.21, 0.36, 0.45, 0.54, and 0.59, respectively. Statistical correlation analysis for bagasse indicated a significant positive relationship between composite thickness and sound reduction coefficient (R^2^ = 0.98). Thus, it can be concluded that increasing the thickness of sound-absorbing materials leads to higher sound reduction coefficients.

**Fig. 2:**
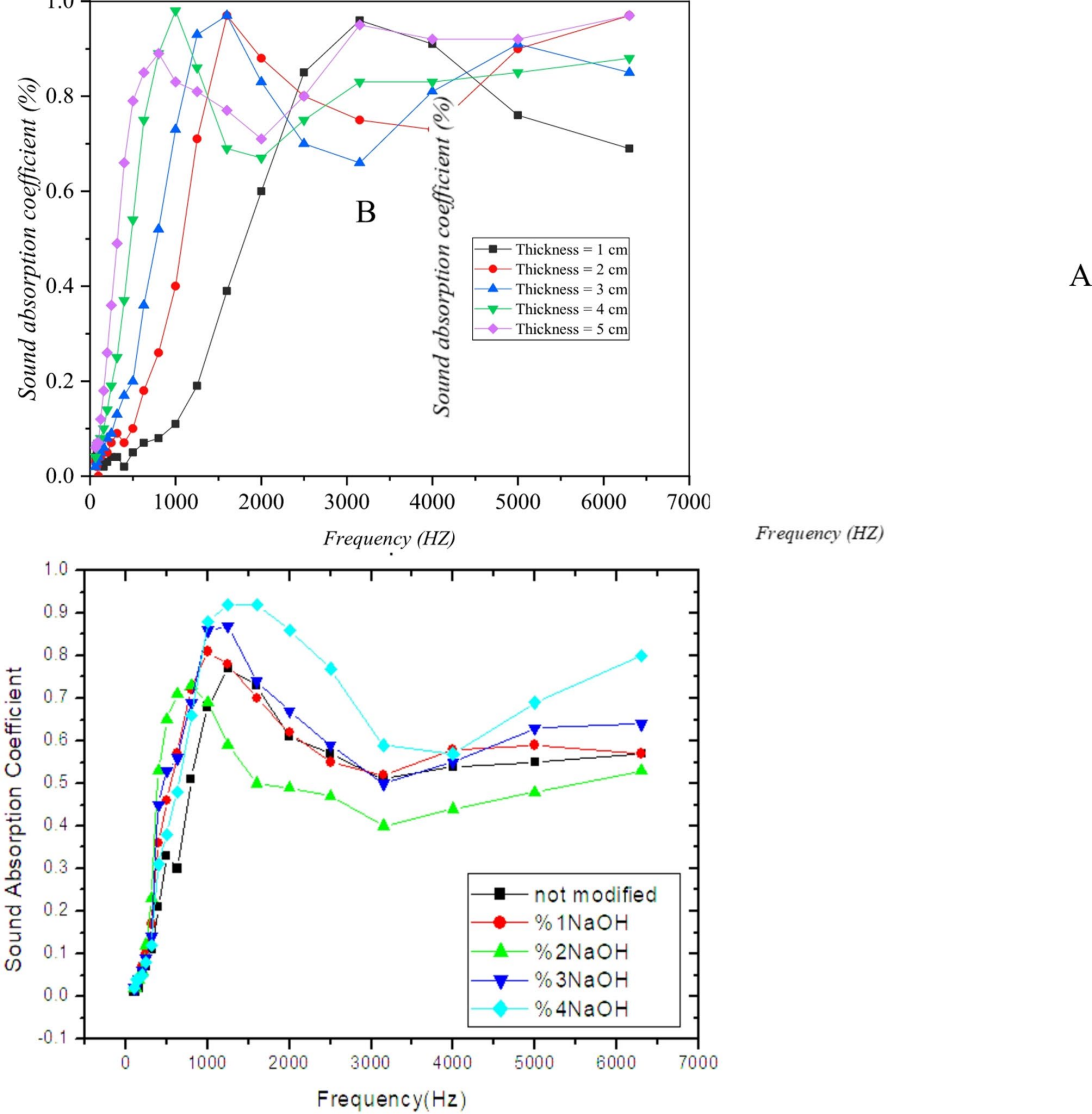
The sound absorption coefficient of bagasse modified with (**A**) NaOH at various concentrations (with an immersion time of 24 h) and (**B**) various thicknesses.

The results of the sound absorption coefficient analysis for bagasse modified with NaOH at different concentrations (with a soaking time of 24 h) are shown in Fig. 2. The findings reveal that soaking bagasse in NaOH solution for 24 h at various concentrations enhances sound absorption in higher frequencies. Based on this study, there is a direct relationship between NaOH concentration and sound absorption coefficient.

**Fig. 3:**
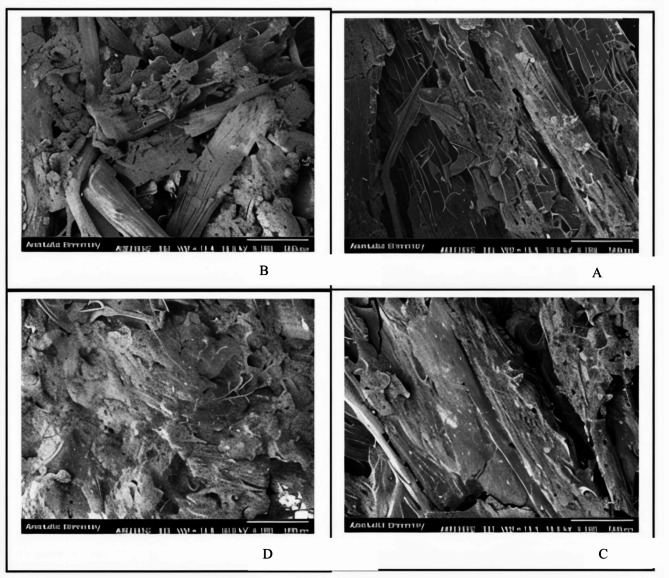
SEM images of bagasse treated with NaOH solution: (**A**) 1% concentration, (**B**) 2% concentration, (**C**) 3% concentration, and (**D**) 4% concentration for a duration of 2 h.

To examine the properties of bagasse fibers, SEM images were analyzed, as shown in Fig. 3. These SEM images were captured using a scanning electron microscope (SEM) (Hitachi S-4160).The image of bagasse, as shown below, reveals a typical structure due to the presence of chemical compounds. These components contribute to the mechanical and thermal strength of the biocomposite. The microscopic structure of bagasse, visible in Fig. 2, indicates that the core is primarily composed of vascular bundles, sclerenchyma cells, and parenchyma.

### The effect of chemical modification of bagasse using NaOH at 1%, 2%, 3%, and 4% concentrations over different immersion times

Immersion of bagasse treated with 1% NaOH solution at various immersion times led to an increase in the sound absorption coefficient at higher frequencies. Moreover, there is a direct correlation between immersion time in 1% NaOH solution and sound absorption coefficient (*p* < 0.05). However, for the 2% NaOH solution, the results indicated that immersion of bagasse did not have a consistent effect on the sound absorption coefficient. At 2 and 13 h of immersion, the sound absorption coefficient increased, but it decreased at other times, showing no direct relationship between immersion time and sound absorption coefficient.

Immersion in 3% NaOH solution also resulted in an improvement in sound absorption at higher frequencies. The evaluation of 4% NaOH solution revealed that this concentration increased the sound absorption coefficient across all frequencies.

### Sound absorption coefficient of Bagasse treated with NaOH at different concentrations

The results of this study indicate that soaking bagasse in NaOH solution for 24 h significantly enhances its sound absorption coefficient, particularly at higher frequencies. A direct correlation was observed between NaOH concentration and sound absorption performance, with higher concentrations generally leading to improved absorption.

At 1000 Hz, which is a crucial frequency range for speech intelligibility and noise control in buildings, the sound absorption coefficient increased from 0.68 (untreated bagasse) to 0.88 when treated with 4% NaOH. Similarly, at 1250 Hz, the absorption reached 0.92, the highest recorded value in this study. The optimal concentration for achieving maximum absorption was 4% NaOH, which showed the most consistent improvements across mid-to-high frequencies. However, at lower frequencies (below 500 Hz), the treatment had less impact, with values ranging between 0.01 and 0.07, indicating that the material remains more effective in mid-to-high frequency noise control.

These findings suggest that NaOH-treated bagasse fibers can effectively enhance sound absorption, making them a sustainable alternative for mid-to-high frequency noise reduction applications such as indoor acoustic panels, automotive sound insulation, and industrial noise control.

**Fig. 4:**
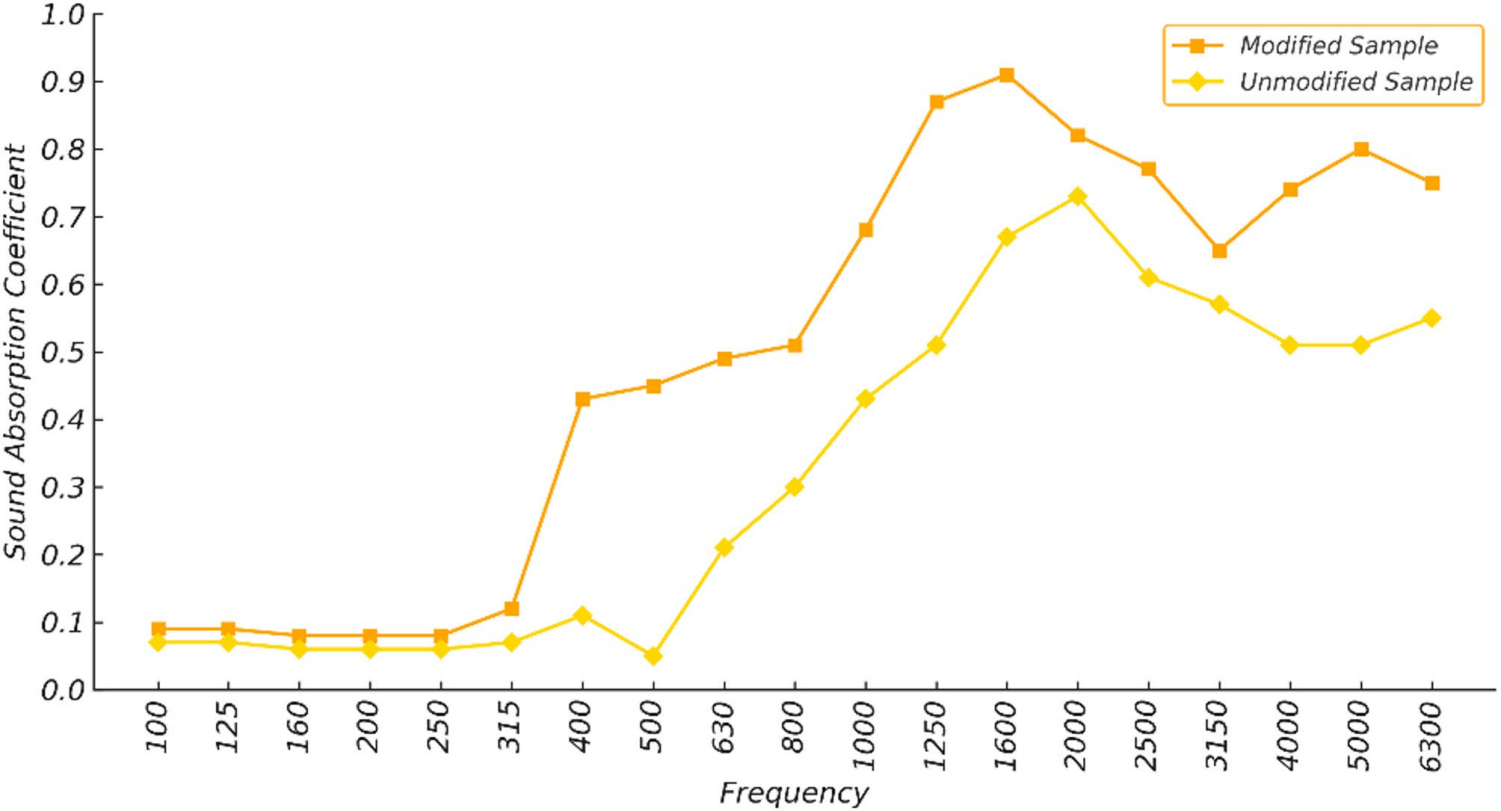
Comparison of sound absorption coefficients of optimally treated bagasse fibers and untreated fibers.

Figure 4 compares the sound absorption coefficients of optimally treated (a 3% concentration and 8-hour immersion time) and untreated (raw) bagasse fibers as measured by an impedance tube. Across the low, mid, and high frequency ranges of one-third octave bands, the optimally treated sample consistently exhibited higher sound absorption than the untreated sample.

It is evident that alkaline treatment increases the SAA index. Specifically, the SAA value rises from its initial levels (before alkaline treatment) of 0.068, 0.553, and 0.558 at low, mid, and high frequencies, respectively, to 0.132, 0.655, and 0.755 after alkaline treatment. In other words, the optimal alkaline treatment results in an increase of 94.11%, 18.44%, and 35.3% in the SAA index at low, mid, and high frequencies, respectively.

### Optimization and modeling of sound absorption coefficient of bagasse treated with NaOH

The required number of experiments was determined based on the level of variables for optimization using a full factorial design. Table 1 presents the matrix used in this study. The experiments were conducted randomly to minimize systematic errors, and the average sound absorption coefficient for each experiment is provided in Table 1.

**Table 1:** Experimental conditions based on a full factorial design.

Test number	NaOH concentration (wt/vol%)	Immersion time (h)	Density (kg/m^3^)	Porosity of fiber’s surface (%)	Thickness (cm)	Avg. absorption (low frequencies)	Avg. absorption (mid frequencies)	Avg. absorption (high frequencies)
1	2	24	150	24.65	2.5	0.14	0.645	0.468
2	2	13	150	24.14	2.5	0.07	0.55	0.691
3	1	18.5	150	35.54	2.5	0.08	0.58	0.573
4	1	7.5	150	30.9	2.5	0.13	0.655	0.755
5	2	13	150	24.14	2.5	0.07	0.55	0.691
6	3	7.5	150	37.39	2.5	0.132	0.655	0.755
7	2	13	150	24.14	2.5	0.07	0.55	0.691
8	2	13	150	24.14	2.5	0.07	0.55	0.691
9	2	2	150	25.83	2.5	0.104	0.71	0.698
10	4	13	150	49.04	2.5	0.122	0.696	0.741
11	2	13	150	24.14	2.5	0.07	0.55	0.691
12	0	0	150	44.6	2.5	0.068	0.553	0.558
13	3	18.5	150	22.4	2.5	0.081	0.573	0.573

### Optimization and modeling results of sound absorption coefficient of bagasse treated with NaOH

The optimization and modeling results of the sound absorption coefficient of NaOH-treated bagasse at low, mid, and high frequencies, based on the RSM method, are presented below. The findings indicate that treated bagasse fibers exhibit better sound absorption at high frequencies compared to low frequencies. Statistical analysis also revealed a significant correlation between NaOH concentration, immersion time, and sound absorption coefficient. The highest SAC index was observed when the NaOH concentration was set to 3% and the immersion time was 7.5 h. Accordingly, increasing NaOH concentration and immersion time up to specific limits (3% concentration, 7.5-hour immersion) resulted in the desired response, i.e., an increase in the average sound absorption coefficient.

The statistical parameters, including sum of squares, degrees of freedom, mean squares, and F and p values for the proposed model, are presented in Table 2. In the provided formula, “A” represents the NaOH concentration, and “B” represents the immersion time. The analysis results, along with other statistical parameters, are displayed in Table 3. However, to enhance the model’s validity, the AB interaction factor was excluded.

**Table 2:** Analysis of variance (ANOVA) with the exclusion of factor AB.

Source	Sum of squares	Degrees of freedom (DF)	Mean square	F value	*p* value
Model	0.0394	5	0.0079	9.51	0.0050
A	0.0065	1	0.0065	7.83	0.0266
B	0.0069	1	0.0069	8.28	0.0237
A^2^	0.0082	1	0.0082	9.86	0.0164
B^2^	0.0237	1	0.0237	28.55	0.0011
Residual	0.0058	7	0.0008		
Total	0.0452	12			

Table 3 presents the models obtained for different frequency ranges. In the data analysis based on the RSM method, a quadratic model was suggested to determine the SAC for low and mid-frequency ranges. For the high-frequency range, the software proposed both a linear model and a quadratic equation. However, considering the p-value, the linear model was selected.

**Table 3:** Models obtained for different frequency ranges.

Frequency	Model type	Proposed model
Low	quadratic	SAC = + 0.0746 + 0.0092 A − 0.0024B − 0.0002AB + 0.0065 A^2^ + 0.0133B^2^
Mid	quadratic	SAC = + 0.5542 + 0.0232 A − 0.0239B + 0.0289 A^2^ + 0.0321B^2^
High	Linear	SAC = + 0.6597 + 0.0305 A − 0.0687B

### Thermal insulation properties

The results of the investigation into the effect of soaking bagasse in NaOH solution at different concentrations are presented in Table 4. According to the findings, the thermal conductivity of untreated bagasse was reported to be 0.095 W/(m^2^ K). The results of soaking bagasse in NaOH solution with varying concentrations showed that initially, an increase in NaOH concentration slightly reduced the thermal conductivity of the bagasse, but it subsequently increased to some extent. According to the Romanian Norm C107/2-2005 standard^41^. a material with thermal conductivity less than 0.065 W/(m^2^ K) and thermal resistance greater than 0.50 (m^2^ K)/W can be considered a thermal insulator. Therefore, based on the results of this study, none of the materials tested can be classified as thermal insulators.

**Table 4:** Results of thermal conductivity analysis of Bagasse soaked in NaOH solution at different concentrations.

Thermal conductivity (W/(m^2^ K))	Bagasse sample
0.095	Without NaOH
0.091	1% Concentration
0.107	2% Concentration
0.127	3% Concentration
0.108	4% Concentration

### Surface porosity measurement

The surface porosity of both raw bagasse and NaOH-treated bagasse was measured under various treatment conditions. Each experimental run recorded the NaOH concentration (in weight% per volume), the soaking time (in hours), and the resulting porosity percentage. Statistical analysis revealed that there was no significant relationship between either the NaOH concentration or the soaking time and the porosity percentage (*P* > 0.05). For instance, one run with a 2% NaOH solution and a 24 h soaking time yielded a porosity of 65.24%, while another run under the same 2% concentration but with a 13 h soaking time resulted in a porosity of 14.24%. Other conditions included treatments with 1%, 3%, and 4% NaOH over soaking times ranging from 2 to 24 h, producing porosity values between 4.22% and 83.25%. Overall, these results indicate that the treatment parameters do not have a significant impact on the surface porosity of the bagasse samples.

## Discussion

Alkaline chemical modification is one of the most common methods for treating natural fibers. This process disrupts hydrogen bonds in the fiber network, removing lignin, waxes, and oils from the outer surface of the cell wall, ionizing the hydroxyl groups in the fibers, and polymerizing cellulose into alkoxide. The results of this study showed that sodium hydroxide alters the structure of bagasse fibers, and there is a direct relationship between NaOH concentration and fiber diameter. According to studies, alkaline treatment with sodium hydroxide reduces fiber diameter, improves adhesion and fungal resistance, and enhances the quality of the fibers^42^.

According to the results of this study, the peak sound absorption of bagasse composites occurs in the frequency range of 1000 to 2000 Hz. The findings of Somaei et al. showed that alkaline treatment, by increasing surface roughness, enhances the amount of cellulose on the fiber surface and improves fiber interlocking. This increases the number of possible reaction sites, which, in turn, enhances the mechanical properties of lignocellulosic natural fibers^43^.

As mentioned earlier, removing materials from the fiber surface reduces fiber diameter. A smaller fiber diameter provides a larger contact surface and a more tortuous path, which increases airflow resistance through the composites made of natural fibers. This, in turn, enhances sound absorption in the low-frequency range. Increased resistance to airflow also dissipates sound energy through friction between sound waves and air molecules, further improving sound absorption at low frequencies^44^. Accordingly, the results of the present study are consistent with those of the study by Somaii et al.

The results indicate that NaOH solution at concentrations above 4% destroys the parenchyma tissue of bagasse. Parenchyma is the simplest plant tissue, characterized by thin and uniform cell walls. Parenchyma tissues contain cells with thin, permeable primary cell walls that are metabolically active. Parenchyma is defined by its thin, uniform primary cell walls and the absence of secondary cell walls, whereas sclerenchyma is distinguished by thick secondary cell walls within the primary cell walls. Due to this feature, sclerenchyma cells are easily identifiable. Sclerenchyma tissues have thick cell walls that provide structural rigidity and elasticity to plants.

The primary difference between parenchyma and sclerenchyma is the presence of secondary cell walls in sclerenchyma cells. The primary cell wall, due to its permeability, allows small molecules to enter the cell and facilitates the extraction of chemically modified substances from the cell body. These cells are often referred to as chlorenchyma because of their ability to perform photosynthesis, a process in which water, carbon dioxide, and light easily enter the cells to produce sugars that plants use as an energy source. Additionally, parenchyma cells are adapted for storing specific substances in plants.

Sclerenchyma cells produce lignin, a substance that hardens the matrix of the cell wall, resulting in a highly rigid secondary wall that resists decay. Lignin prevents water from penetrating the cell walls, which can lead to cell death if the entire cell is covered. To prevent this, the secondary cell walls of sclerenchyma have small tunnels connecting neighboring cells. These pits act as pathways for water and nutrients.

The chemical structure and morphology of bagasse and sugarcane fibers are different. Fibers have thick cell walls that contribute to tissue strength, whereas parenchyma cells are very small and have extremely thin walls, which reduce their mechanical strength. This difference affects their acoustic and thermal properties^45^.

The results of examining the effect of soaking bagasse in sodium hydroxide solution at concentrations ranging from 1 to 4% showed that increasing the sodium hydroxide concentration up to 3% and immersion time to 7–8 h enhances the sound absorption coefficient. However, further increases in sodium hydroxide concentration and immersion time reduce the sound absorption coefficient, and at concentrations above 4%, producing an absorbent material becomes infeasible, as the composite takes on a doughy consistency.

This phenomenon might be due to the reaction between PVA adhesive and sodium hydroxide, which destroys the fibrous structure of bagasse. Therefore, the SAC index reaches its maximum when the sodium hydroxide concentration is around 3% and the immersion time is approximately 7.5 h. This result aligns with the findings of the study conducted by Somaii et al.^44^.

As demonstrated in this study, chemical modification (alkaline treatment) using sodium hydroxide increases the sound absorption coefficient of natural composites made from bagasse fibers within the one-third octave band frequency range. The composite optimized for alkaline treatment increased the SAA index by 94.11%, 18.44%, and 35.3% in low, mid, and high frequencies, respectively^44^. Similar improvements have been observed in other recent studies on chemically treated natural fibers—such as Sansevieria, Hibiscus, and banana stem—where liquid smoke or NaOH–silane treatments enhanced tensile strength, crystallinity, and thermal stability. These parallels highlight the broader potential of surface modifications in improving the performance of bio-based composites^6,7,46,47^. The study also revealed that increasing the thickness of the absorbent material enhances the sound absorption coefficient. This increase in sound absorption may result from the extended dissipation process through thermal conductivity and viscosity interactions between air and the absorbent materials within the composite, which is amplified by the greater thickness of the composite.

Additionally, the findings showed that as the thickness of the absorbent material increases, the peak frequency shifts to lower frequencies. Therefore, increasing the thickness of the absorbent material improves its performance at lower frequencies. Consistent with these findings, Xie and colleagues also observed that with increased thickness, the porosity and absorption coefficient of the materials improve^48^. Acoustic tests conducted on palm tree fibers and oil palm fibers have also shown that increasing the thickness of the absorbent layer leads to a higher sound absorption coefficient. At the same time, the peak sound absorption coefficient shifts toward lower frequencies^49^. According to the results of this study, the thermal conductivity of untreated bagasse was reported as 0.095 W/(m^2^ K). The results of soaking bagasse in sodium hydroxide solution at various concentrations showed that increasing the sodium hydroxide concentration initially caused a slight decrease in the thermal conductivity of bagasse, followed by a gradual increase. standard A material with a thermal conductivity of less than 0.065 W/(m^2^ K) can be considered a thermal insulator according to the Romanian Norm C107/2-2005 standard^50^. Therefore, based on the results of this study, none of the materials reported can be classified as thermal insulators. Similarly, in the study by Asdrubalil et al., it was suggested that the thermal conductivity of insulating materials should be less than 0.07 W/(m^2^ K)^51^.

In the present study, predictive models for the sound absorption coefficient have been demonstrated. Many studies propose empirical relationships as predictive models for the sound absorption coefficient to achieve the best alignment between experimentally measured values and the optimization of predicted sound absorption coefficients. Theoretical models provided for analytical investigation, considering certain primary physical properties of materials such as porosity, curvature, and airflow resistance, calculate the energy consumption of sound within sound-absorbing materials and predict other acoustic behaviors in porous sound absorbers^44^. In Gaur’s study, the use of bagasse as a substitute for fiberglass for sound control was also proposed^52^.

## Conclosion

In conclusion, the optimization of the alkaline treatment process for natural fibers to achieve the maximum sound absorption coefficient (SAC) in bagasse fiber composites occurs when sodium hydroxide concentration is set at 3% and immersion time at 7.5 h. Under these optimal conditions, the SAC value is significantly improved, especially in the 1000–2000 Hz frequency range, making the treated bagasse a promising sustainable acoustic material.

This study demonstrates that alkaline treatment with sodium hydroxide effectively enhances the acoustic properties of bagasse-based natural fiber composites. This optimized treatment method enhances fiber–matrix compatibility and overall mechanical, thermal, and acoustic performance, meeting the growing demand for eco-friendly materials.

While most conventional sound-absorbing materials in buildings, such as fiberglass and mineral wool, offer effective noise reduction, they are often associated with health risks and environmental concerns due to the release of formaldehyde and volatile organic compounds (VOCs)^50^. Treated bagasse fibers offer a sustainable, biodegradable, and cost-effective alternative to synthetic sound absorbers, making them suitable for various industrial applications. The findings of this study emphasize the potential of NaOH-treated bagasse fibers in residential, commercial, and industrial environments, where their lightweight nature, affordability, and eco-friendly properties can be beneficial. These fibers can be integrated into building applications, such as wall and ceiling panels in offices, conference rooms, and recording studios, to enhance indoor acoustic performance. Additionally, they can be utilized in the automotive and transportation sectors, including car door panels, railway cabins, and aircraft interiors, to effectively reduce noise pollution. In industrial environments, bagasse-based composites can function as acoustic panels for noise control in manufacturing plants and factories. Thermal insulation remains limited but could improve by combining bagasse with foams, multilayer systems, or other bio-based reinforcements. As the demand for green building materials increases, there is a growing need for biodegradable and low-carbon alternatives that reduce environmental impact. This research lays the groundwork for scaling up the use of treated bagasse fibers in the construction industry, helping to minimize agricultural waste and reduce dependence on non-renewable resources. Future work should focus on hybridizing bagasse fibers, assessing long-term durability under real conditions, and benchmarking performance against conventional insulators.

Ultimately, the integration of sustainable materials such as NaOH-treated bagasse fibers presents a promising path toward the development of high-performance, environmentally friendly sound absorbers. As renewable and eco-efficient materials, they support global sustainability and industrial innovation.

### Recommendations

Given the degradation of the bagasse fiber structure at sodium hydroxide concentrations above 4%, it is recommended that future studies explore concentrations below 1%. Additionally, due to the reduction in sound absorption coefficients at immersion times exceeding 8 h, it is advisable to avoid immersing fibers in sodium hydroxide solutions for durations longer than this in subsequent research.

## Data Availability

The data supporting the findings of this study are included within the manuscript and the supplementary information files. For any additional information or specific requests regarding the dataset, interested parties are encouraged to contact the third author, Mehrana Esnaasharieh, via email at mehranaesnaasharieh@gmail.com.

## References

[1] TG, Y. G. et al. Biopolymer-based composites: an eco-friendly alternative from agricultural waste biomass. *J. Compos. Sci.* **7**(6), 242 (2023).

[2] Sangmesh, B. et al. Development of sustainable alternative materials for the construction of green buildings using agricultural residues: A review. *Constr. Build. Mater.* **368**, 130457 (2023).

[3] Thapliyal, D. et al. Natural fibers composites: origin, importance, consumption pattern, and challenges. *J. Compos. Sci.* **7**(12), 506 (2023).

[4] Castañeda-Niño, J. P., Mina-Hernandez, J. H. & Solanilla-Duque, J. F. Effect of cellulose nanofibers and plantain Peel fibers on mechanical, thermal, physicochemical properties in bio-based composites storage time. *Results Eng.* 104185 (2025).

[5] Ibrahim, F. H., Setiawan, R. A., Steven, S. & Mardiyati, Y. Towards sustainable composites: fabrication, characterization, and biodegradation of All-Cellulose composites (ACC) from Ramie (Boehmeria nivea) and Luffa (Luffa cylindrica). *Results Eng.* 104695 (2025).

[6] Wirawan, W. A., Sabitah Ay, Choiron, M. A., Muslimin, M., Zulkarnain, A. & Budiarto, B. W. Effect of chemical treatment on the physical and thermal stabillity of Hibiscus Tiliaceus bark Fiber (HBF) as reinforcement in composite. *Results Eng.* **18**, 101101 (2023).

[7] Muslimin, M. et al. Enhancement of Sansevieria Trifasciata Laurentii Fiber properties with liquid smoke treatment. *J. Nat. Fibers*. **22**(1), 2453482 (2025).

[8] Shafiee, S. A., Imran, S. N. M. & Zaki, Z. Z. M. An approach utilizing varied sugarcane Bagasse densities as biobased acoustic panels for educational institutions. *Int. J. Bus. Technol. Manage.* **6**(S1), 52–62 (2024).

[9] Khosro, S. K. et al. Acoustical, thermal, and mechanical performance of Typha Latifolia fiber panels: experimental evaluation and modeling for sustainable Building applications. *J. Building Eng.* **99**, 111579 (2025).

[10] Srisawas, M., Kerdkaew, T. & Chanlert, P. From invasive species to bio-based composites: utilizing water hyacinth for sound absorption and insulation. *Ind. Crops Prod.* **220**, 119242 (2024).

[11] Mohammadi, M. et al. Recent progress in natural fiber reinforced composite as sound absorber material. *J. Building Eng.* 108514 (2024).

[12] Hemmati, N. et al. Acoustic and thermal performance of wood strands-rock wool-cement composite boards as eco-friendly construction materials. *Constr. Build. Mater.* **445**, 137935 (2024).

[13] Wirawan, W. A., Choiron, M. A., Siswanto, E. & Widodo, T. D. Morphology, structure, and mechanical properties of new natural cellulose fiber reinforcement from Waru (Hibiscus tiliaceus) bark. *J. Nat. Fibers*. **19**(15), 12385–12397 (2022).

[14] Mizoue, T., Miyamoto, T. & Shimizu, T. Combined effect of smoking and occupational exposure to noise on hearing loss in steel factory workers. *Occup. Environ. Med.* **60**(1), 56–59 (2003). 12499458 10.1136/oem.60.1.56 PMC1740373

[15] Chraif, M. The effects of radio noise in multiple time reaction tasks for young students. *Procedia-Social Behav. Sci.* **33**, 1057–1062 (2012).

[16] Alimohamadi, I., Soltani, R., Azkhosh, M., Gohari, M. & Moosavi, B. Study of role extroversion of caused by traffic noise on mental function of the students. *Iran. Occup. Health*. **7**(4), 7–0 (2011).

[17] Aliabadi, M., Mahdavi, N., Farhadian, M. & Shafie Motlagh, M. Evaluation of noise pollution and acoustic comfort in the classrooms of Hamadan university of medical sciences in 2012. *Iran. J. Ergon.* **1**(2), 19–27 (2013).

[18] Bellelli, F., Arina, R. & Avallone, F. On the impact of operating condition and testing environment on the noise sources in an industrial engine cooling fan. *Appl. Acoust.* **227**, 110252 (2025).

[19] Arjunan, A., Baroutaji, A., Robinson, J., Vance, A. & Arafat, A. Acoustic metamaterials for sound absorption and insulation in buildings. *Build. Environ.* 11250 (2024).

[20] Clark, C. & Stansfeld, S. A. The effect of transportation noise on health and cognitive development: A review of recent evidence. *Int. J. Comp. Psychol.* ;**20**(2) (2007).

[21] Hahad, O. et al. Noise and mental health: evidence, mechanisms, and consequences. *J. Expo. Sci. Environ. Epidemiol.* 1–8 (2024). 10.1038/s41370-024-00642-5 PMC11876073 38279032

[22] Bluhm, G., Nordling, E. & Berglind, N. Road traffic noise and annoyance-An increasing environmental health problem. *Noise Health*. **6**(24), 43–49 (2004). 15703140

[23] Ghanbarzadeh Alamdari, Z., Khavanin, A. & Kokabi, M. Manufacturing sound absorber based on combined recycling of polyethylene trephetalat and polystyrene at low and median frequencies. *Bimon. Audiology-Tehran Univ. Med. Sci.* **17**(1), 1–10 (2008).

[24] Ersoy, S. & Küçük, H. Investigation of industrial tea-leaf-fibre waste material for its sound absorption properties. *Appl. Acoust.* **70**(1), 215–220 (2009).

[25] Chis, T. V. et al. Integrated noise management strategies in industrial environments: A framework for occupational safety, health, and productivity. *Sustainability* **17**(3), 1181 (2025).

[26] Zhao, X-D., Yu, Y-J. & Wu, Y-J. Improving low-frequency sound absorption of micro-perforated panel absorbers by using mechanical impedance plate combined with Helmholtz resonators. *Appl. Acoust.* **114**, 92–98 (2016).

[27] Bhingare, N. H., Prakash, S. & Jatti, V. S. A review on natural and waste material composite as acoustic material. *Polym. Test.* **80**, 106142 (2019).

[28] Chenzhi, C. & Mak, C. M. Noise Attenuation capacity of a Helmholtz resonator. *Adv. Eng. Softw.* **116**, 60–66 (2018).

[29] Lv, L. et al. Effect of micro-slit plate structure on the sound absorption properties of discarded corn cob husk fiber. *Fibers Polym.* **16**, 1562–1567 (2015).

[30] Cobo, P. & de Espinosa, F. M. Proposal of cheap microperforated panel absorbers manufactured by infiltration. *Appl. Acoust.* **74**(9), 1069–1075 (2013).

[31] Arenas, J. P. & Ugarte, F. A note on a circular panel sound absorber with an elastic boundary condition. *Appl. Acoust.* **114**, 10–17 (2016).

[32] Cao, L., Fu, Q., Si, Y., Ding, B. & Yu, J. Porous materials for sound absorption. *Compos. Commun.* **10**, 25–35 (2018).

[33] Xinzhao, X., Guoming, L., Dongyan, L., Guoxin, S. & Rui, Y. Electrically conductive graphene-coated polyurethane foam and its epoxy composites. *Compos. Commun.* **7**, 1–6 (2018).

[34] Berardi, U. & Iannace, G. Predicting the sound absorption of natural materials: Best-fit inverse laws for the acoustic impedance and the propagation constant. *Appl. Acoust.* **115**, 131–138 (2017).

[35] Sarja, A. *Editor Integrated Life Cycle Design of Materials and Structures* (CIB World Congress, 1998).

[36] Ashour, T., Georg, H. & Wu, W. Performance of straw Bale wall: A case of study. *Energy Build.* **43**(8), 1960–1967 (2011).

[37] Cascone, S. M., Cascone, S. & Vitale, M. Building insulating materials from agricultural by-products: A review. *Sustainability in Energy and Buildings: Proceedings of SEB 2019* 309–318 (2020).

[38] Martellotta, F., Cannavale, A., De Matteis, V. & Ayr, U. Sustainable sound absorbers obtained from Olive pruning wastes and Chitosan binder. *Appl. Acoust.* **141**, 71–78 (2018).

[39] Oldham, D. J., Egan, C. A. & Cookson, R. D. Sustainable acoustic absorbers from the biomass. *Appl. Acoust.* **72**(6), 350–363 (2011).

[40] Glé, P., Gourdon, E. & Arnaud, L. Acoustical properties of materials made of vegetable particles with several scales of porosity. *Appl. Acoust.* **72**(5), 249–259 (2011).

[41] Dénes, T-O. et al. Analysis of sheep wool-based composites for Building insulation. *Polymers* **14**(10), 2109 (2022). 35631991 10.3390/polym14102109 PMC9143407

[42] Kalia, S., Kaith, B. & Kaur, I. Pretreatments of natural fibers and their application as reinforcing material in polymer composites—a review. *Polym. Eng. Sci.* **49**(7), 1253–1272 (2009).

[43] Samaei, S. E., Asilian Mahabadi, H., Mousavi, S. M., Khavanin, A. & Faridan, M. Effect of alkali treatment on diameter and tensile properties of Yucca Gloriosa fiber using response surface methodology. *J. Nat. Fibers*. **19**(7), 2429–2442 (2022).

[44] Samaei, S. E., Mahabadi, H. A., Mousavi, S. M., Khavanin, A. & Faridan, M. Optimization and sound absorption modeling of Yucca Gloriosa natural fiber composites. *Iran. Occup. Health*. **18**(1), 1–17 (2021).

[45] Sanjuan, R., Anzaldo, J., Vargas, J., Turrado, J. & Patt, R. Morphological and chemical composition of pith and fibers from Mexican sugarcane Bagasse. *Holz Als Roh-und Werkst.* **59**, 447–450 (2001).

[46] Muslimin, M. et al. Effect of liquid smoke treatment on banana stem fibers as composite reinforcement. *SAINSTECH NUSANTARA*. **2**(1), 1–11 (2025).

[47] Sulistyo, A. & Wirawan, W. Evaluation of tensile strength and flexural strength of GFRP composites in different types of matrix polymers. *J. Achievements Mater. Manuf. Eng.* **123**(2), 49–57 (2024).

[48] Xie, Z. K., Ikeda, T., Okuda, Y. & Nakajima, H. Characteristics of sound absorption in lotus-type porous magnesium. *Jpn. J. Appl. Phys.* **43**(10R), 7315 (2004).

[49] Abd ALRahman, L., Raja, R. I., Rahman, R. A. & Ibrahim, Z. Comparison of acoustic characteristics of date palm fibre and oil palm fibre. *Res. J. Appl. Sci. Eng. Technol.* **7**(8), 1656–1661 (2014).

[50] Tămaş-Gavrea, D-R. et al. A novel acoustic sandwich panel based on sheep wool. *Coatings* **10**(2), 148 (2020).

[51] Asdrubali, F., D’Alessandro, F. & Schiavoni, S. A review of unconventional sustainable Building insulation materials. *Sustainable Mater. Technol.* **4**, 1–17 (2015).

[52] Gaur, M., Muzammil, M. & Khan, A. A. Bagasse: A replacement of glass wool as an acoustic material. *Ergonomics in Caring for People: Proceedings of the International Conference on Humanizing Work and Work Environment 2015* (Springer, 2018).

